# Supplementary material for: Combining Nanopore Sequencing with Recombinase Polymerase Amplification Enables Identification of Dinoflagellates from the Alexandrium Genus, Providing a Rapid, Field Deployable Tool
Source: Toxins (Basel). 2023 Jun 1;15(6):372. doi: 10.3390/toxins15060372 (PMC10302762; doi:10.3390/toxins15060372)
Supplement: Supplementary file 1 [file toxins-15-00372-s001.zip › Supplemantary_material/S4_Correlation_graphs.pptx]

## Slide 1
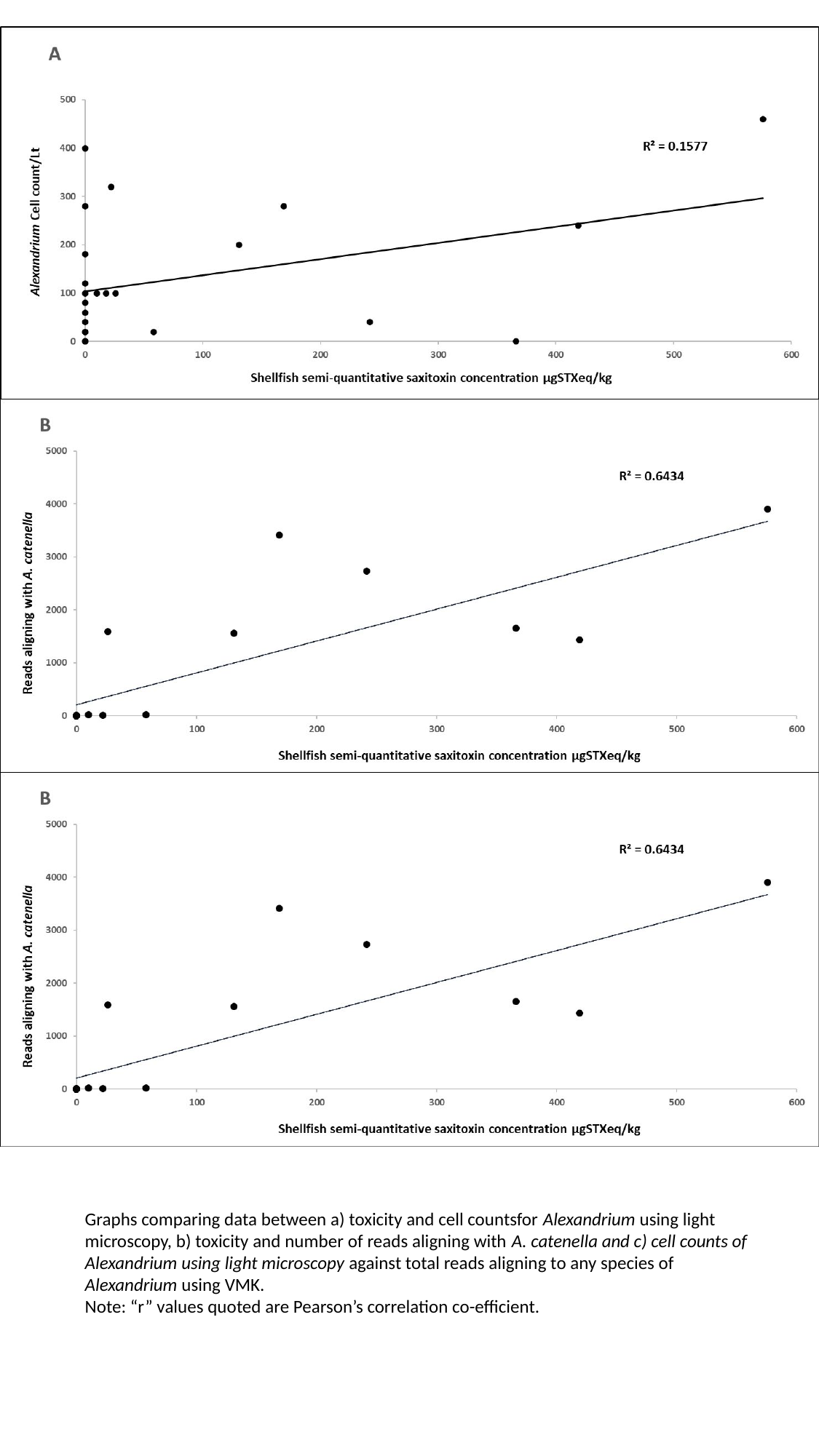

Graphs comparing data between a) toxicity and cell countsfor Alexandrium using light microscopy, b) toxicity and number of reads aligning with A. catenella and c) cell counts of Alexandrium using light microscopy against total reads aligning to any species of Alexandrium using VMK.
Note: “r” values quoted are Pearson’s correlation co-efficient.
