# Supplementary figures and images for: Combining Nanopore Sequencing with Recombinase Polymerase Amplification Enables Identification of Dinoflagellates from the Alexandrium Genus, Providing a Rapid, Field Deployable Tool
Source: Toxins (Basel). 2023 Jun 1;15(6):372. doi: 10.3390/toxins15060372 (PMC10302762; doi:10.3390/toxins15060372)

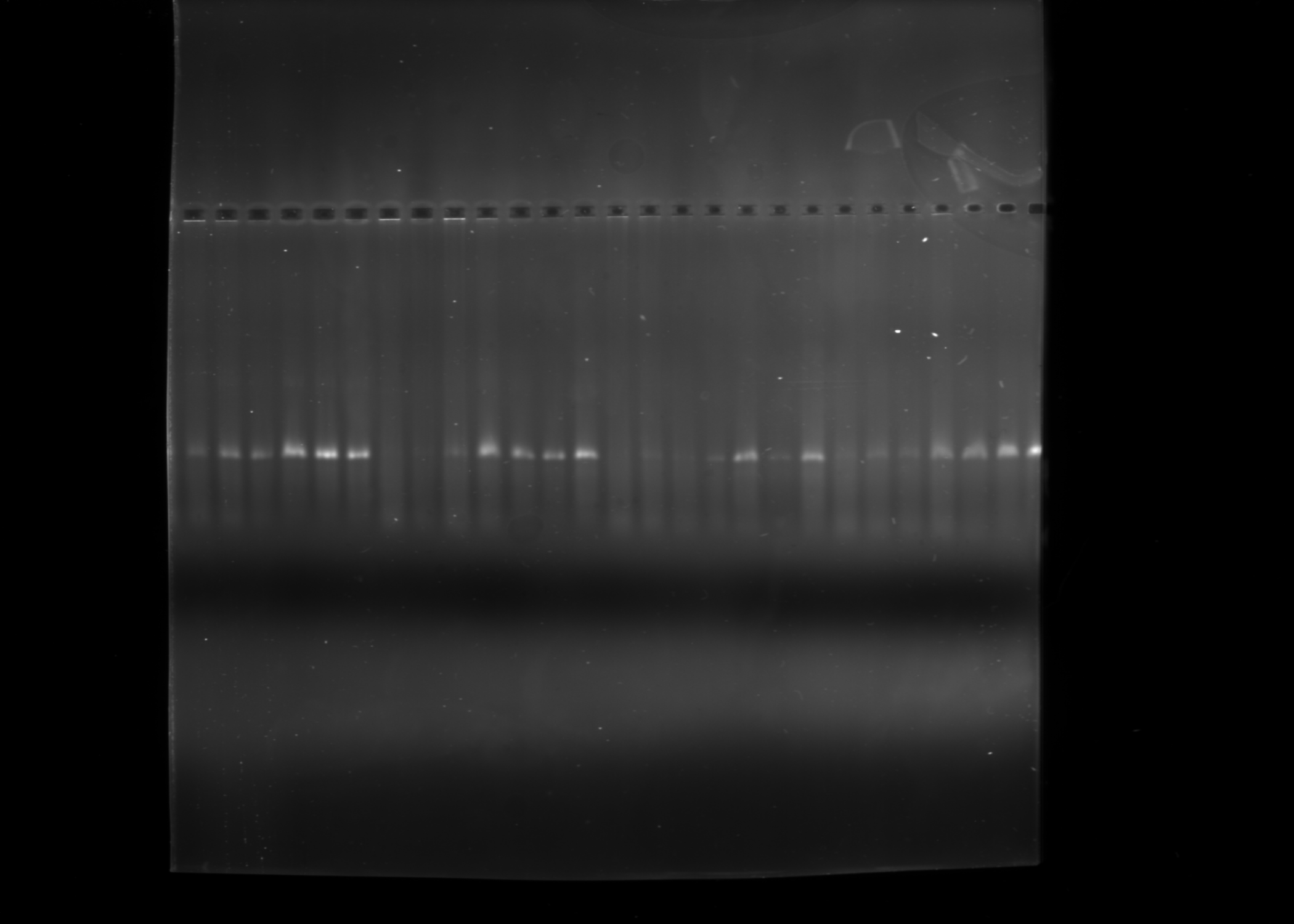

Supplement: Supplementary file 1 [file toxins-15-00372-s001.zip › Supplemantary_material/S1_Gels/primer_selection_1.tif]

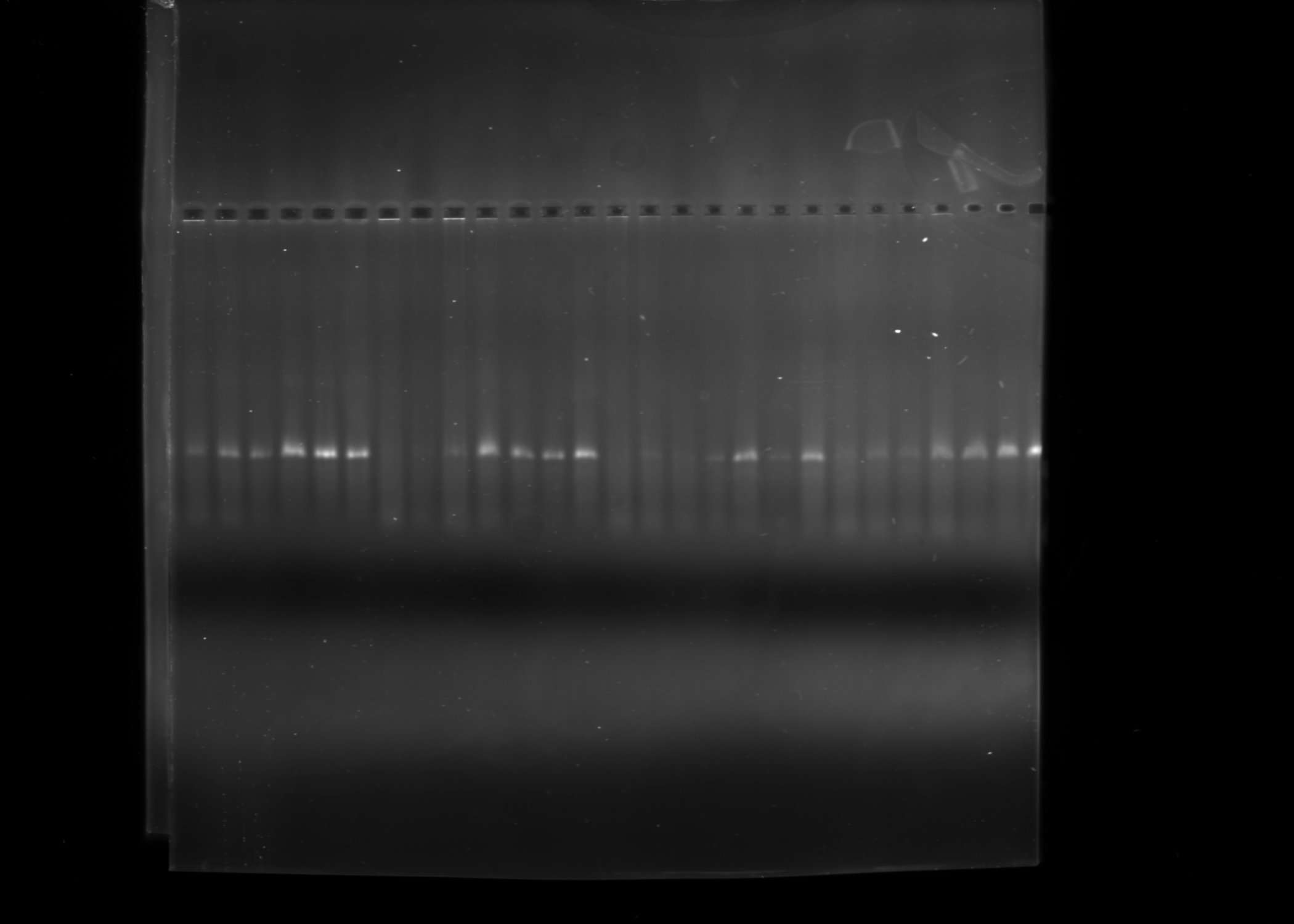

Supplement: Supplementary file 1 [file toxins-15-00372-s001.zip › Supplemantary_material/S1_Gels/Primer_selection_2.tif]

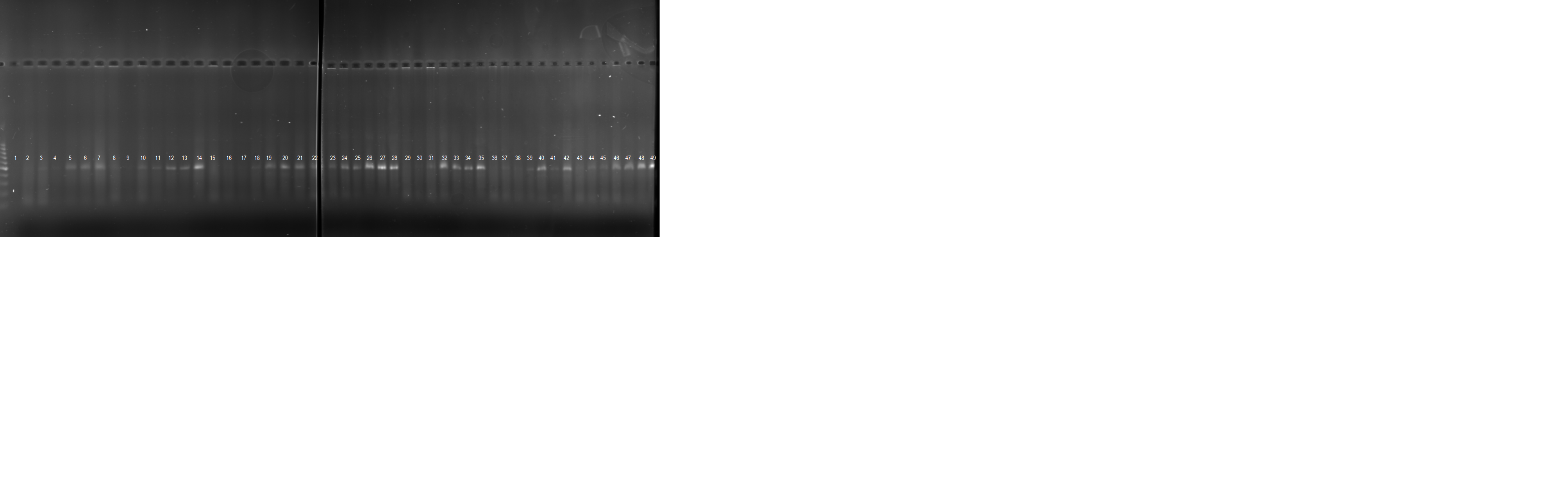

Supplement: Supplementary file 1 [file toxins-15-00372-s001.zip › Supplemantary_material/S1_Gels/Primer_selection_gel_annotated.png]

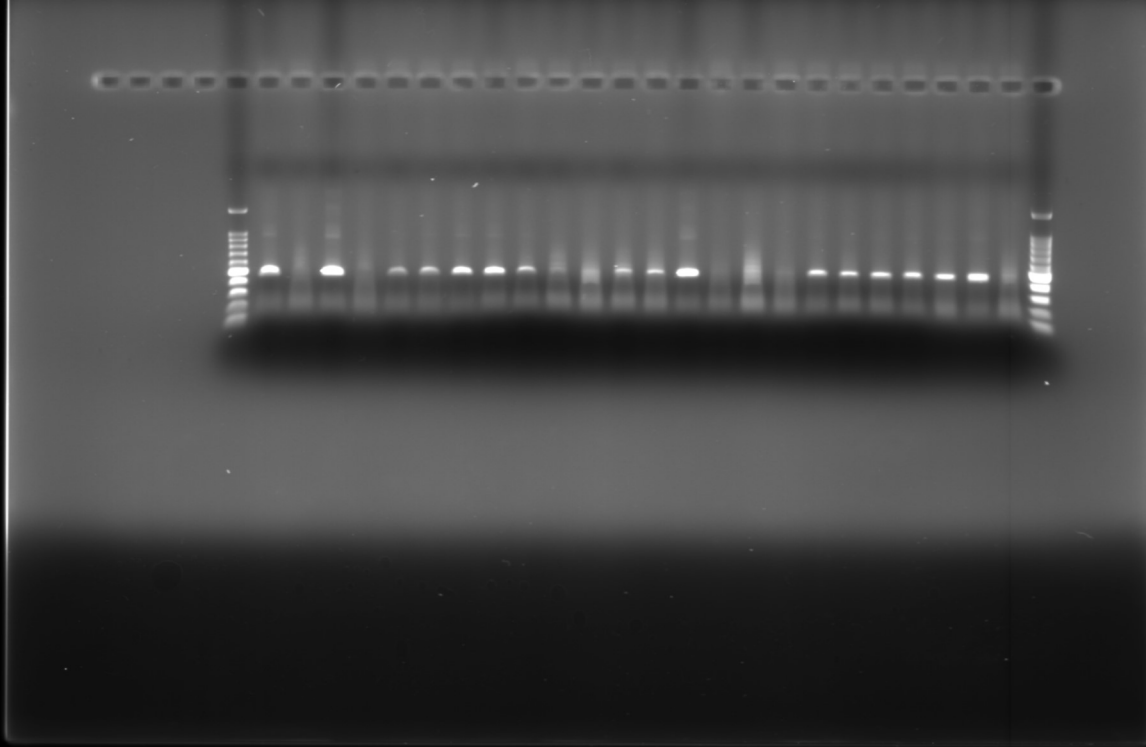

Supplement: Supplementary file 1 [file toxins-15-00372-s001.zip › Supplemantary_material/S1_Gels/Shetland_Samples_RPA.pdf]

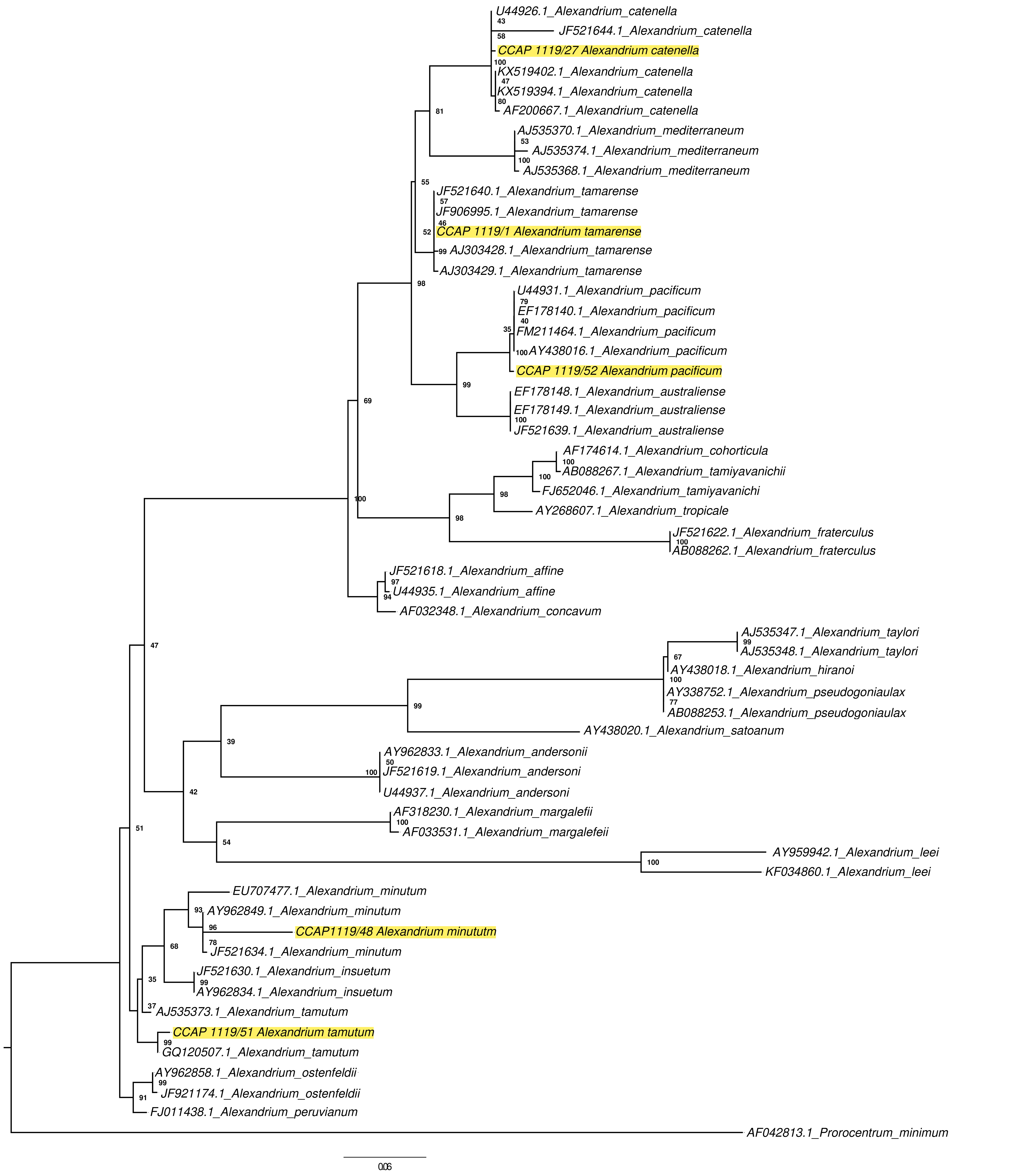

Supplement: Supplementary file 1 [file toxins-15-00372-s001.zip › Supplemantary_material/S3_phylo_tree_branch_lengths.png]

## Slide 1
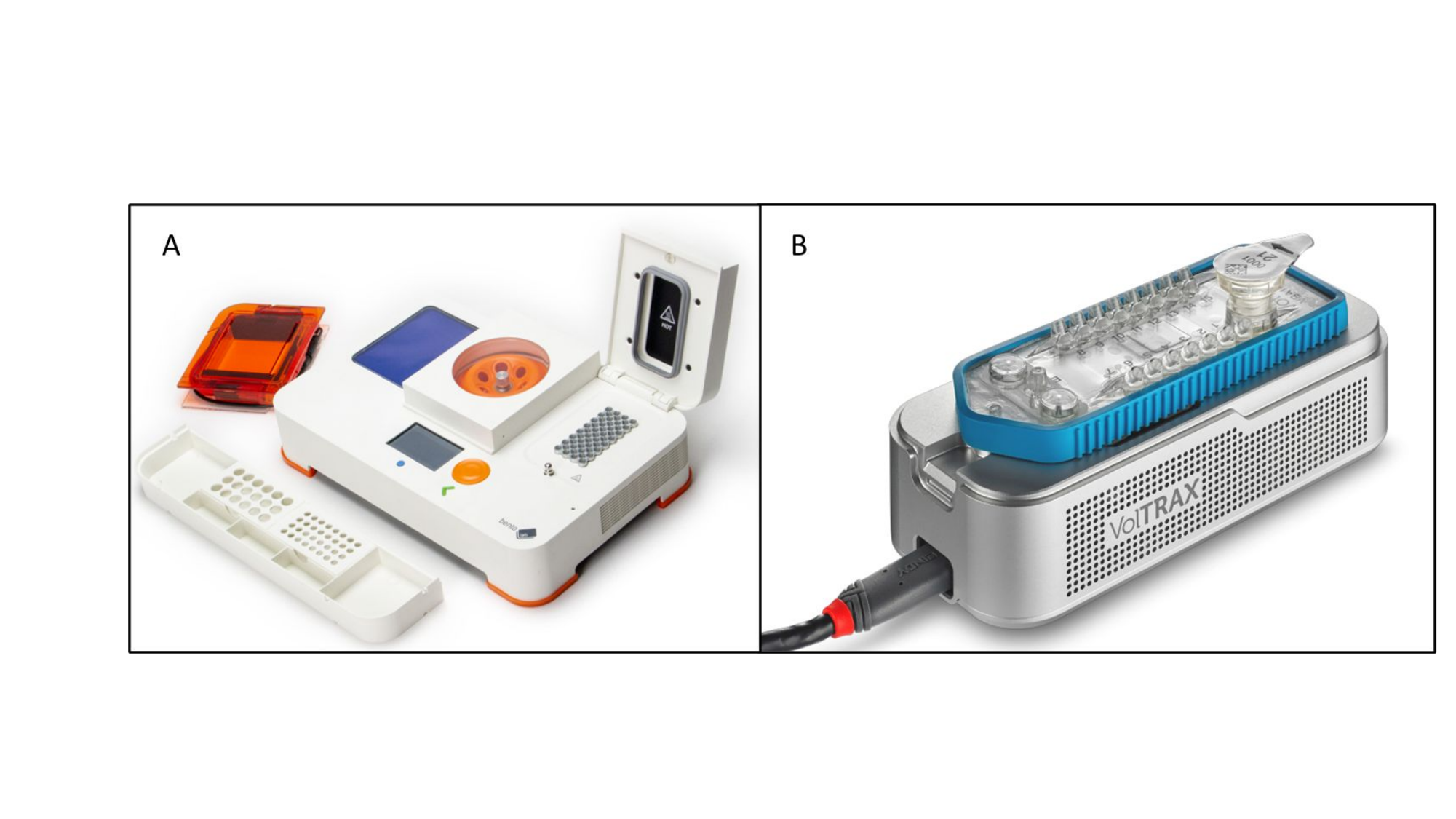

Supplement: Supplementary file 1 [file toxins-15-00372-s001.zip › Supplemantary_material/S6_VolTRAX_&_Bentolab.pptx]
